# The First *Paenibacillus larvae* Bacteriophage Endolysin (PlyPl23) with High Potential to Control American Foulbrood

**DOI:** 10.1371/journal.pone.0132095

**Published:** 2015-07-13

**Authors:** Ana Oliveira, Marta Leite, Leon D. Kluskens, Sílvio B. Santos, Luís D. R. Melo, Joana Azeredo

**Affiliations:** CEB—Centre of Biological Engineering, University of Minho, 4710–057, Braga, Portugal; ContraFect Corporation, UNITED STATES

## Abstract

Endolysins, which are peptidoglycan-degrading enzymes expressed during the terminal stage of the reproduction cycle of bacteriophages, have great potential to control Gram-positive pathogens. This work describes the characterization of a novel endolysin (PlyPl23) encoded on the genome of *Paenibacillus larvae* phage phiIBB_Pl23 with high potential to control American foulbrood. This bacterial disease, caused by *P*. *larvae*, is widespread in North America and Europe and causes important economic losses in apiculture. The restriction to antibiotic residues in honey imposed by the EU legislation hinders its therapeutic use to combat American foulbrood and enforces the development of alternative antimicrobial methods. The new endolysin described herein has an *N*-acetylmuramoyl-*L*-alanine amidase catalytic domain and exhibits a broad-spectrum activity against common *P*. *larvae* genotypes. Moreover, the enzyme displays high antimicrobial activity in a range of pH that matches environmental conditions (pH between 5.0 and 7.0), showing its feasible application in the field. At pH 7.0, a concentration of 0.2 μM of enzyme was enough to lyse 10^4^ CFU.mL^-1^ of *P*. *larvae* in no more than 2 h. The presence of sucrose and of the substances present in the larvae gut content did not affect the enzyme activity. Interestingly, an increase of activity was observed when PlyPl23 was previously incubated in royal jelly. Furthermore, *in vivo* safety evaluation assays demonstrated that this enzyme is not toxic to the bee larvae. The present work describes for the first time an endolysin encoded in a *P*. *larvae* phage that presents high potential to integrate a commercial product to control the problematic American foulbrood.

## Introduction

Endolysins (lysins) are phage-encoded enzymes that are expressed at the end of the phage life cycle in the infected bacteria to allow the release of the newly assembled virions. These enzymes target and cleave bonds of the cell wall peptidoglycan, degrading the murein layer. Despite this activity exerted from within the host, the high efficiency of the endolysins when added externally to Gram-positive cells was already reported, being already described as the most powerful biological antimicrobials, just comparable with chemical agents [1].

Lysins from phages infecting Gram-positive bacteria have a typical modular structure, being composed of at least two clearly distinct functional domains, the catalytic domain and the cell wall binding domain (CBD) separated by a binding region (linker). A sequence comparison among enzymes of the same class has shown that the catalytic domain is a conserved region, while the CBDs are much more variable [2]. Generally, these enzymes are very specific due to the presence of the CBD that targets specific bonds of the cell wall surface. So far, no resistance mechanisms or resistant phenotypes were detected after endolysin exposure probably because the lysins’ targets are essential for bacterial viability [3]. These facts make endolysins promising strategies to control Gram-positive pathogens. *Paenibacillus larvae* (*P*. *larvae*) are Gram-positive bacteria that cause a severe disease in larvae of *Apis mellifera* and other *Apis* spp, usually known as American Foulbrood (AFB) [4]. A common strategy for the prevention and treatment of affected colonies is the use of antibiotics, particularly oxytetracycline hydrochloride [5]. However, the great concern regarding bacterial resistance [6] and the accumulation of chemical residues in honey, decreasing the quality and hindering the marketing, dampen the use of antibiotics in beekeeping industry. Indeed, in the European context, the use of antibiotics in beekeeping is not allowed (Regulation (EEC) 2377/90 and further amendments). Furthermore, at highly infectious scales, millions of spores drive disease transmission within and between colonies and to achieve an effective treatment the colony has to be burned. This causes important economic losses in the sector. Consequently, the urgent need to develop new effective methods against AFB is unquestioned.

We have previously isolated and reported the first known *P*. *larvae* phage genome [7] and the *in silico* analysis enabled the identification of its predicted endolysin. In the work described herein we characterized and assessed the potential of the heterologously expressed endolysin, PlyPl23, in the control of AFB, through the *in vitro* evaluation of its antimicrobial ability against *P*. *larvae* and *in vivo* evaluation of its safety in bee larvae.

## Materials and Methods

### Isolation of *Paenibacillus larvae* strains

*P*. *larvae* strains were isolated from 42 samples derived from honey, wax and brood samples from Portuguese hives with clinical symptoms of AFB. Samples were pre-treated depending on the matrix. The isolation of *P*. *larvae* from honey was adapted from Genersch and Otten [8]. Briefly, 1 g of honey was diluted 1:1 (w/v) in double distilled water (ddH_2_O), homogenized overnight at 37°C and heated at 90°C, 6 min (for spore-activation). For the treatment of brood samples, dead larvae were collected form brood combs with a sterile swab, emulsified in 500 μL ddH_2_O and the resultant suspension was heated at 90°C, 6 min. Wax samples (1.5 g) were dissolved in 10 mL benzene (1:10 w/v), then 2 mL of the resultant suspension was diluted 1:4 (v/v) in ddH_2_O, and the aqueous phase was recovered [9]. After treating each type of sample, 100 μL of the obtained suspension was sewed in MYPGP agar (10 g.L^-1^ Mueller-Hinton, 15 g.L^-1^ yeast extract, 3 g.L^-1^ K_2_HPO_4_, 1 g.L^-1^ Na-pyruvate, 20 g.L^-1^ agar, and 2% glucose). Plates were incubated at 37°C under 5% CO_2_ and evaluated for bacterial growth after 3 to 6 days [10].

### 16S-PCR identification of *P*. *larvae* and rep-PCR analysis

Isolates from field samples were identified as *P*. *larvae* based on a positive PCR result of the 16S rRNA gene. The primers used (Table 1) amplified a 1106 bp fragment [11].

**Table 1:** Primers and PCR conditions used for *P*. *larvae* 16S-PCR identification, rep-PCR analysis and PlyPl23 cloning.

Amplicon	Sequence (5’- 3’)	Annealing (oC.s-1)	Extension (s)
16S rRNA	F: CTTGTGTTTCTTTCGGGAGACGCC; R: TCTTAGAGTGCCCACCTCTGCG	55/30	90
Rep-PCR	ERIC2: AAGTAAGTGACTGGGGTGAGC; ERIC1R: ATGTAAGCTCCTGGGGATTCA	47/45	120
PlyPl23	Ply23F: AGAAGCCATATGATGGAAATCAGACAGATGCTAGTAGGA (*Nde*I); Ply23R: TACGATGGATCCTCACAGGCACCCACTCGCCAAAA (*Bam*HI)	53/30	60

The bacterial DNA was purified using the NucleoSpin Tissue kit (Macherey-Nagel) and amplified using Kapa*Taq* (Kapa Biosystems) according to the manufacturer’s instructions. The PCR product was analyzed on a 1% agarose gel stained with SYBR Safe (NZYTech) and visualized under UV light.

Typing of *P*. *larvae* strains was performed through genomic fingerprinting. Rep-PCR (repetitive sequence-based PCR), was carried as reported by Genersch and Otten (2003)[8]. The PCR was done in the same conditions as previously, using the primers described in Table 1. The PCR product was analyzed on a 2% agarose gel, as described above.

### Bioinformatics analysis of the phage phiIBB_Pl23 endolysin gene

The annotation of the *Siphoviridae P*. *larvae* phage phiIBB_Pl23 identified gp21 as an endolysin protein, designated as PlyPl23 [7]. *In silico* analysis was carried out by searching the non-redundant protein database using BLASTP [12] with the amino acid sequence of PlyPl23 as query (GenBank accession number KF010834-21). Functional domains were searched against the Pfam database [13], and the HHpred [14, 15]. The presence of a signal peptide was searched using SignalP 4.0 [16]. The lysin molecular weight (MW), isoelectric point (pI) and charge were predicted using Compute pI/Mw tool, available at http://web.expasy.org/compute_pi/. The identification of the putative amidase catalytic sites was performed using the CDD database [17].

### Cloning, expression, and purification of PlyPl2

Using specific primers (Table 1), the endolysin ORF was amplified by PCR through Kapa*Taq* (Kapa Biosystems) with the phage DNA as template (purified with the GRS Viral DNA/RNA Purification Kit, Grisp) according to manufacturer’s instructions. The PCR product was confirmed on a 0.8% agarose gel.

The amplified gene was cloned into pET28a (Novagen), Kan^R^, conserving the plasmid N-terminal hexahistidine (His)-tag sequence, and used to transform *E*. *coli* JM109 cells. The correct insertion of the gene was confirmed by sequencing and the construct was then cloned into competent *E*. *coli* BL21 (DE3). Expression of the recombinant PlyPl23 in *E*. *coli* BL21 (DE3) was induced with 0.5 mM isopropyl-b-D-thiogalactopyranoside (IPTG) (Fisher Scientific) when cells reach an OD_620nm_ of 0.5 (grown at 37°C, 120 rpm, in a Biosan ES-20/60 orbital shaker–incubator), followed by an overnight incubation at 16°C, 150 rpm, in a Panasonic MIR-S100 orbital shaker. Bacterial cells were harvested with centrifugation at 4 000 x *g* for 20 min, and the pellet was suspended in 1/10 volumes of lysis buffer (20 mM Na_2_H_2_PO_4_, 500 mM sodium chloride, pH 7.4). Afterwards, 10 mM imidazole (Fisher Scientific) was added and the cell disruption was completed by thaw-freezing (3 cycles, from -80°C to room temperature) followed by a 5 min sonication (Cole-Parmer Ultrasonic Processor) for 10 cycles (30 s pulse, 30 s pause), 30% amplitude. Insoluble cell debris was removed by centrifugation (9 500 x *g*, for 30 min, 4°C) and the supernatant was filtered through 0.22 μm PES membrane. Purification was done through 1 mL Ni-NTA agarose stacked in a Polypropylene column (Qiagen), previously equilibrated with 10 mM imidazole, making use of the protein His-tag. The washing step was performed using protein-dependent imidazole concentrations (the lysis buffer was supplemented with 20 mM imidazole in the first washing and 40 mM imidazole in the second washing), and then the sample was eluted in a 300 mM imidazole lysis buffer solution. Samples of each fraction (0.5 mL) were analyzed by SDS-PAGE.

The buffer was changed to the storage buffer (20 mM Tris, pH 8.0, 30% glycerol) using the centrifugal filters Amicon Ultra—0.5 mL (Millipore). The purified protein was stored at -20°C in the same buffer.

### Lytic activity assay

The PlyPl23 lytic activity was determined by measuring the OD_620_ of the bacterial suspension in a Multiskan FC spectrophotometer (Thermo Fisher) or by direct CFU counting. Initially, *P*. *larvae* cells were exponentially grown (OD_620_ = 0.6–0.8) in MYPGP broth, harvested (10 000 x *g*, 5 min), washed twice and suspended in the reaction buffer, Tris 20 mM pH7, alone or supplemented, depending on the assay. In a 96-well plate, a volume providing 2.0 μM of the dialyzed endolysin, which was set as the optimal activity on OD_620_ reduction tests, was added to 100 μL of bacterial suspensions. In the control, the volume of endolysin was substituted by its storage buffer. Before each reading, the plate was shaken for 60 s to homogenize the suspension. Except for the lytic spectra, all the tests were performed with the *P*. *larvae* strain Pl02-23, the host used for phiIBB_Pl23 amplification [7]. The reactions were performed at 37°C, which is similar to the hive temperature [18], for 60 min or 120 min. The lytic activity was calculated according to Eq 1.

$$\frac{\left(r_{t0}-r_{t120})-(c_{t0}-c_{t120}\right)}{c_{t0}}\;*\;100$$ (1)

Where, *r* and *c* are the suspension OD_620_ of the reaction and control respectively and *t0* and *t120* are the samples at time zero min and 120 min respectively. The relative lytic activity was obtained, calculating the ratio between the lytic activity of each test and the higher lytic activity obtained in the experiment. To evaluate the PlyPl23 effect by direct CFU enumeration, the total volume per reaction was 200 μL, in a 96-well plate. The dialyzed endolysin was added at the desired concentration in the reaction tests and its volume substituted by the storage buffer, in the control tests. After incubation at 37°C, 10 μL of enzyme-treated and non-treated bacterial suspensions were collected at 0, 30, 60 and 120 min post-reaction. The suspensions were serial diluted and 100 μL were plated on MYPGP agar. At the end of the reaction (120 min), 100 μL of the remaining volume were plated with no dilutions, to increase the detection limit of the assay. The lytic activity was calculated as mentioned above but using the values of CFU.mL^-1^ instead of OD_620_. All experiments were executed in at least three independent assays.

#### Effect of pH

The optimal pH for the endolysin reaction was tested by assessing the OD_620_ variation, after adding PlyPl23 to *P*. *larvae* strain Pl02-23 suspensions at pH levels ranging from 2.0 to 10.0, in a 1.0 step. The different pH solutions were prepared in Universal buffer (150 mM KCl, 10 mM KH_2_PO_4_, 10 mM Na-citrate and 10 mM H_3_BO_4_), adjusted with HCl or NaOH [19].

#### Effect of ionic strength

For a better assessment of the enzyme performance in the field conditions, these tests were performed at the pH levels of 5.0, 6.0 and 7.0 (larvae and honey bee intestine). For each pH, the influence of NaCl on PlyPl23 lytic activity was evaluated (measuring the OD_620_ variation) by adding different concentrations of NaCl: 0, 100 and 200 mM.

#### PlyPl23 lytic spectra

A total of 23 *P*. *larvae* strains and 4 negative control strains (*Bacillus cereus*—CEB collection, *Bacillus subtilis*—DSMZ 10), *Lactobacillus paracasei*—CECT 277 and *Lactobacillus pentosus*—CECT 4023) were used to evaluate the lytic spectra of PlyPl23, by assessing the OD_620_ variation. The endolysin was added to each strain, previously suspended in Tris pH 7.0 (20 mM). The lytic spectrum obtained for the phage phiIBB_Pl23 was performed against the same strains to compare the antimicrobial effect. This was achieved by adding a drop of 10 μL of phage (10^8^ PFU.mL^-1^) to the formerly tested bacteria. For that, strains were grown until mid-log phase as described and 100 μL of each suspension were incorporated in 3.0 mL MYPGP 0.5% agar. After dried the plates, the phage was spotted on the lawn. After an overnight incubation at 37°C and 5% CO_2_, the presence of lysis zones were indicative of strain sensitivity to phiIBB_Pl23.

#### Enzyme concentration and in vitro antimicrobial activity assay

The assessment of the optimal enzyme concentration to reduce *P*. *larvae* until non-detectable levels was done by direct CFU enumeration, with 0.2 and 2.0 μM PlyPl23 and 10^4^, 10^5^ and 10^6^ CFU.mL^-1^ initial bacteria concentration. For that, exponentially grown (OD_620_ ≈ 0.6) Pl 02–23, suspended in Tris pH 7.0 (20 mM), 200 mM NaCl, was diluted 10^2^, 10^3^ and 10^4^-fold in the same buffer. For each bacterial suspension, the enzyme was added at both indicated concentrations and the activity was calculated as described above.

#### Effect of sucrose, larval gut content and royal jelly

The effect of several conditions regarding the practical application of PlyPl23 activity was evaluated. To assess the larval gut content effect, worker larvae from an apiary with colonies of *Apis mellifera iberiensis*, located in the north of Portugal (Famalicão), were gently grafted with a larvae-picking tool (1^st^ and 2^nd^ instar in one group; 3^rd^ and 4^th^ instar in the other group). The larvae were weighted, suspended in NaCl 0.9% (1:1 w/v) and homogenized with glass beads by vortexing for 2 min. The resultant suspension was filtered through 0.22 μm PES membrane. This was called “homogenized larvae” (HL) and was assumed to contain the substances present in larvae gut content to which the endolysin would be exposed in field conditions. The *P*. *larvae* strain Pl02-23 was suspended in 100 μL HL and 2.0 μM PlyPl23 was added to the test sample. The effect of HL on the endolysin activity was evaluated by measuring the OD_620_ variation.

The evaluation of the royal jelly (RJ) effect on PlyPl23 activity was made to mimic the likely contact with a similar product in which larvae from the first instars are floating [20], and that might happen after spray administration of the enzyme to brood in field conditions. RJ was purchased from a Portuguese supplier (Apiguarda), and after confirming the absence of *P*. *larvae* spores, as described above for honey, it was prepared for tests through dilution (1:1 w/v) in ddH_2_O. Before the activity tests, PlyPl23 was diluted (1:10 v/v) in the prepared RJ suspension to a final concentration of 20 μM and incubated for 1 h at 37°C. After, 20 μl of the resultant suspension was added to 180 μl of 10^7^ CFU.mL^-1^ *P*. *larvae* (Pl02-23), previously suspended in Tris pH 7.0 (20 mM), 200 mM NaCl (final concentration of 2.0 μM PlyPl23). The RJ effect on PlyPl23 activity was evaluated by CFU counts.

The effect of two different concentrations of sucrose, 25% and 50%, on the enzyme activity was also tested to assess the efficacy of administering the product with sucrose due to its suitability for beekeeper practices. For this, PlyPl23 was previously diluted 1:10 (v/v) in each sucrose concentration (final enzyme concentration of 20.0 μM) and then 10 μL were added to 90 μL of strain Pl02-23, previously suspended in Tris pH 7.0 (20 mM), 200 mM NaCl (final enzyme concentration of 2.0 μM). The activity was evaluated through OD_620_ variation.

Finally, the stability of PlyPl23 on simulated field conditions occurring before larvae ingestion was assessed. Briefly, the enzyme was diluted to a final concentration of 20.0 μM in a suspension composed of 1:1 (w/v) RJ, 50% sucrose (final concentration), 200 mM NaCl, and incubated for 6 h at 37°C. The activity assays were performed by adding 10 μL of the previously incubated solution to 90 μL of a Pl02-23 suspension strain in Tris pH 7.0 (20 mM), 200 mM NaCl. The OD_620_ variation was calculated.

#### Development of resistances in *P*. *larvae* induced by PlyPl23

In order to assess the development of resistances in surviving colonies a procedure based on Schuch et al. [21] was performed with the following modifications. The concentration of endolysin able to inhibit 50% of bacterial isolates (MIC_50_) was previously determined to be 0.2 μM. After that, 100 μL of an overnight suspension of PlyPl23 was incubated for 120 min with 0.2 μM, after which the total volume was plated onto MYPGP agar plates. After an overnight incubation, ten different colonies were individually inoculated in MYPGP broth and incubated overnight. The cultures were regrown until mid-log phase and a volume of 100 μL was used to test susceptibility to 2.0 μM PlyPl23. The OD_620_ variation was monitored for 120 minutes. At the same time, another 100 μL from the same culture were incubated with 0.2 μM of the enzyme (MIC_50_) to induce resistance and the resultant suspensions were plated on MYPGP agar plates. This procedure was repeated ten times resulting in ten cycles of bacteria exposition to the enzyme at MIC_50_ in an attempt to induce resistance to the endolysin.

#### Evaluation of the stability of PlyPl23 and storage conditions

The stability of the endolysin in storage buffer was tested to assess the best storage conditions. The evaluation of the enzyme activity following storage at room temperature (RT), 4°C, and—20°C (supplemented with 30% glycerol), for 22 weeks was monitored periodically. The effect of lyophilization was also evaluated, and the OD_620_ variation was assessed after reconstitution of the lyophilized sample with ddH_2_O. The activity tests were performed with Pl02-23 suspended in 100 μL of the reaction buffer referred above, for 120 min, at 37°C, as described above.

### Effect of PlyPl23 on *P*. *larvae* spores

The effect of PlyPl23 was also assessed on spores and germinating spores of *P*. *larvae*.

#### *P*. *larvae* spores’ preparation

*P*. *larvae* spores’ suspensions were prepared based on the procedure described by Alvarado et al. [22], with some modifications. Briefly, Pl02-23 strain was incubated in MYPGP agar, at 37°C under 5% CO_2_ for 8 days. Ice-cold ddH_2_O water was poured onto bacterial lawns, stored at 4°C for about 5 h, and the suspension was then collected. Spores were pelleted by centrifugation (8 000 x *g*, 5 min) and suspended in fresh ddH_2_0. After five washing steps spores were purified following a methodology described by Sacks and Alderton [23]. In this procedure an aqueous two-phase system distribution, composed of potassium phosphate (PB)-polyethylene glycol (PEG)-water was used. A gradient was created by dissolving 5.6 g of PEG 4000 in 17 mL of 3 M phosphate buffer (pH 7.4), allowing for phase separation. Afterwards, the spores’ suspension was carefully poured on the top layer (total volume of 50 mL) and centrifuged at 1 500 x *g* for 3 min (20°C). Next, the upper phase (where spores were concentrated) was carefully recovered. Spores were washed five times, as described above, and suspended in ddH_2_O.

The effectiveness of the separation procedure and consequent spore purification was determined by microscopy (Olympus BX51, 1000 x magnification), with the staining technique described by Schaeffer and Fulton [24]. Summarizing, to stain endospores, 10 μL of air-dried spore suspension deposited on a glass slide were covered with a blotting paper saturated with malachite green (0.5% (w/v), and placed over a container of boiling water. After 5 min, the slide was washed with ddH_2_O and counterstained with safranin (0.25% w/v) for 30 s, to stain vegetative cells. The slide was washed again with dH_2_O, allowed to dry and examined for the presence of spores (bright green contrasting with red to pink vegetative cells).

#### Activity of PlyPl23 on *P*. *larvae* spores

Spores (OD_580_≈ 0.5) were heat activated (HA) at 70°C for 30 min, and 100 μL were used for each test, performed in a 96-well plate. The following conditions were tested: i) Effect of PlyPl23 on spores suspended in Tris pH 7.0; ii) effect of PlyPl23 on spores suspended in germinants (a 3 mM L-tyrosine – 3 mM uric acid solution, as described by Alvarado et al. [22]); iii) effect of PlyPl23 on spores 2 h after germinants addition. Controls were made by replacing the enzyme by its storage buffer. The OD_580_ variation was monitored for 7 h (every 10 min until 2 h, every 30 min until 4 h and every 60 min until 7 h).

In order to monitor the spores germination state (bright green spores on dormant state contrasting with red to pink spores on germinating state), 10 μL of each sample were collected from reactions at 0 min, 30 min, 2 h and 7 h, and examined by microscopy. Staining and subsequent procedures were performed as described above.

### Toxicity tests in artificially reared larvae

No specific permissions were required to raise honeybee larvae in laboratory conditions. Under the Laboratory Animals Directive of the EU (2010/63/EU), reissued in 2010, experiments with arthropods do not require authorities’ approval. The *in vivo* studies performed in the scope of this work did not involve endangered or protected species.

Despite considering two feasible ways of administering the endolysin, orally to adult bees or spraying directly to bee larvae, the *in vivo* assays described herein were performed to assess the potential toxic effect of the endolysin only to bee larvae. It was assumed that if there were no harmful effects in young larvae, the same would happen in adult bees, which present a more adapted gut.

The artificial diet to feed larvae, obtained from the apiary mentioned above, was prepared according to Aupinel [25], and consisted of 50% (w/v) RJ and 50% of an aqueous solution of 12% (w/v) D-glucose, 12% (w/v) fructose and 2% (w/v) yeast extract. Just before feeding, the sugar solution was mixed with fresh RJ 1:1 (w/w) and warmed to 35°C. Larvae measuring between 1 and 2 mm (major length of the C-shape, here mentioned as “C-length”) were grafted from each comb with a larvae-picking tool and gently deposited on a 96-well plate (one larvae per well) previously filled with 50 μL of diet. The plate was transferred into an exicator containing a saturated solution of K_2_SO_4_ that provided a 96% relative humidity atmosphere (confirmed with a hygrometer), closed hermetically and placed in an incubator at 35°C. In order to prevent fungal growth, a 10% (w/v) ground propolis infusion was prepared in water (based on Pujirahayu et al. [26]), and after an overnight incubation at 40°C, the decanted suspension was diluted 1:4 (v/v) in the used K_2_SO_4_ saturated solution.

A volume of 50 μL of diet was provided to the larvae with a micropipette once a day. After 24 h of grafting (adaptation period) and until the end of the experiment, 13 viable larvae were fed with diet containing 2.0 μM PlyPl23 and other 13 larvae with diet containing buffer Tris pH 8.0 (20 mM) replacing the volume of PlyPl23. Larvae were measured daily for the subsequent 4 days and weighted at the end of the trial (5^th^ day). The variation in the C-length and weight between the 2^nd^ and the 5^th^ day was compared between both groups.

### Statistical analysis

Statistics were calculated by using Prism 5 software (GraphPad, CA, USA). Mean and standard deviations were determined for three independent experiments and results were presented as mean ± SD. The assays described on 2.5.1, 2.5.4, 2.5.5, 2.5.6 and 2.6.2 were compared using one-way analysis of variance (ANOVA) and Bonferroni post-hoc test. The assay described on 2.5.2 was compared using two-way ANOVA and Bonferroni post-hoc test. For all tests a confidence level of 99.9% was used.

Regarding the *in vivo* results, the variability between the sample (measured by the standard deviation) was estimated in 10% based on previous tests with artificially raised larvae, and the minimum difference between the groups considered as meaningful (with a statistical power of 95%) was 15%. Based on these assumptions, the minimum experimental units (larvae) that should be used in this study is 11 per group [27].

## Results

### Identification and expression of the endolysin from phage phiIBB_Pl23

*In silico* analysis identified gp21 as an endolysin, which we have designated PlyPl23. PlyPl23 is composed of 224 residues with a predicted MW of 25.8 kDa, a pI of 6.08 and with no identified signal peptide. The prediction tools BlastP, Pfam and HHpred identified an *N*-acetylmuramoyl-*L*-alanine amidase belonging to the Amidase_2 family (Pfam 01510) at the N-terminus encompassing two third of the protein. CDD database identified 5 putative amidase catalytic residues: two histidines (position 29 and 129), a phenylalanine (position 53), a lysine (position 135) and a cysteine (position 137). The remaining third, the C-terminus did not display homology to any identifiable domain. BlastP analysis showed that the closest relatives to PlyPl23 are five *Bacillus* phage endolysins, with homologies to the N-terminal region, coverage ranging from 66% to 71% and identities of at least 59% to 72% (e-values: 5x10^-63^ to 1x10^-81^). Furthermore, no significant relatives were found among the *Paenibacillus* genus.

PlyPl23 was cloned in pET28a and expressed in *E*. *coli* BL21 (DE3) and further purified using affinity chromatography. SDS-PAGE showed a band near 25.0 kDa corresponding to an overexpressed protein, which was consistent with the calculated molecular weight of the lysin, 25.8 kDa (S1 Fig).

### PlyPl23 Lytic activity

#### Effect of pH

**Fig 1:**
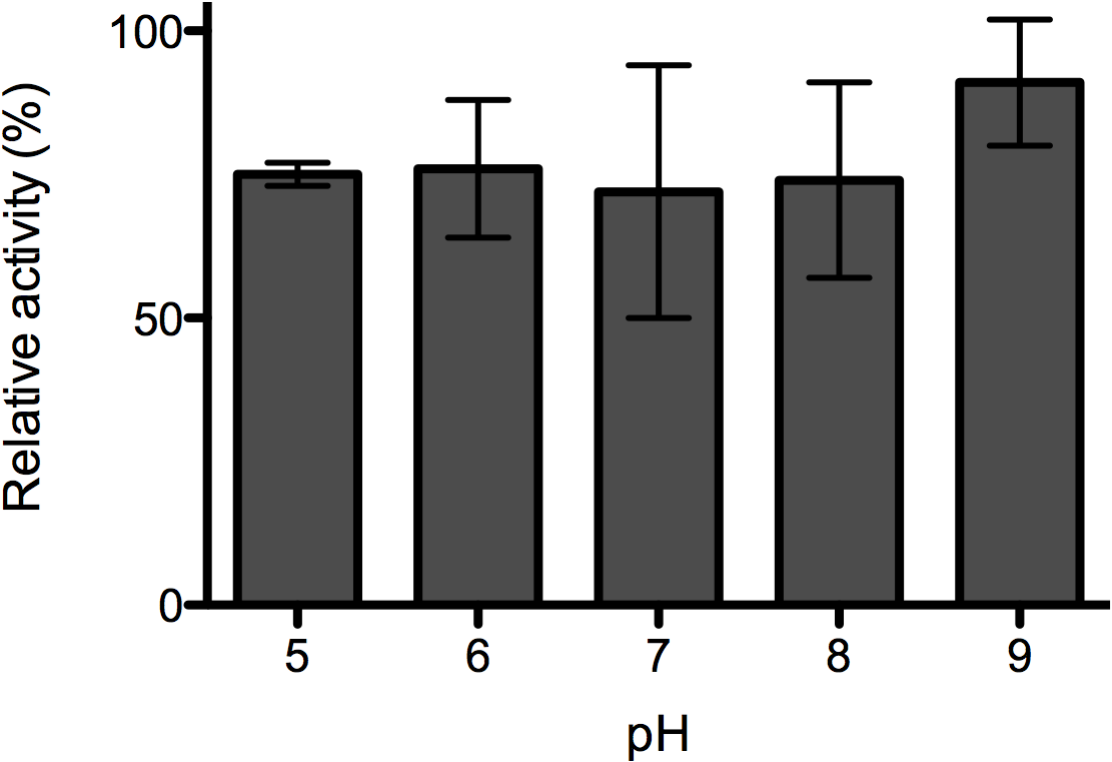
Effect of pH on PlyPl23 lytic activity. PlyPl23 activity on *P*. *larvae* Pl02-23 at different pH values, ranging from 5 to 9. Relative lytic activity was obtained as the ratio between the lytic activity of each test and the highest value obtained (the reference value was 70.9% lytic activity). Each column represents the mean of triplicate experiments, and error bars indicate the standard deviation.

The effect of pH on PlyPl23 lytic activity was investigated using Universal buffer in order to cover a broad range of pH. Results revealed that no differences in PlyPl23 relative activity were observed (p>0.05) between pH 5.0 and 9.0 (Fig 1). At the pH values of 2.0, 3.0, 4.0 and 10.0 it was not possible to recover any bacterial cells in the controls (without endolysin). Consequently, the endolysin activity for these pH values could not be assessed. Based on these results, pH 7.0 was set for the following assays giving its proximity with the larva gut pH [22].

#### PlyPl23 lytic spectrum

The lytic spectrum of PlyPl23 was assessed against a panel of 20 *P*. *larvae* isolates and 4 additional strains belonging to other related bacterial species (*Bacillus cereus*, *Lactobacillus paracasei*, *Lactobacillus pentosus*, *Bacillus subtilis*), and compared with that of the respective phage phiIBB_Pl23. *P*. *larvae* strains were isolated from 42 different environmental samples and belong to ERIC I (16 strains) and ERIC II (4 strains) genotypes (Table 2).

**Table 2:** Lytic spectra of PlyPl23 and the phage phiIBB_Pl23 against 20 *P*. *larvae*, 2 *Bacillus* and 2 *Lactobacillus* strains. NA: Non applicable.

Strain^a^	Source	ERIC genotype	phiIBB_Pl23	PlyPl23
**Pl01- (01, 03, 07, 07b2, 13, 14, 18)**	**honey**	**I**	+	+
**Pl01- (02, 10, 12, 19)**	**honey**	**II**	+	+
**Pl02- (21, 30b, 30c)**	**dead larvae**	**I**	-	+
**Pl02- (23** ^*^ **, 27, 29, 30a, 31)**	**dead larvae**	**I**	+	+
**Pl03-28**	**wax**	**I**	-	+
**Bacillus cereus (CEB Bc-01)**	**dead larvae**	**NA**	-	-
**Lactobacillus paracasei (CECT 277)**	**sour milk**	**NA**	-	-
**Lactobacillus pentosus (DSM 20314)**	**corn silage**	**NA**	-	-
**Bacillus subtilis (DSMZ 10)**	**unavailable**	**NA**	-	-

* Mentioned as H23 in Oliveira *et al*. (2013).

^a^ Criteria of the strain nomenclature—species: *Paenibacillus larvae* (Pl); source: 01—honey; 02—dead larvae; 03- wax; reference of the isolate: number between brackets.

Results showed that while the phage phiIBB_Pl23 was able to lyse 16 of the 20 strains (80%), its endolysin was active against all the tested *Paenibacillus larvae* strains. The *Bacillus* and *Lactobacillus* strains were not lysed by the phage or by the enzyme.

#### Effect of ionic strength

Different concentrations of NaCl (0, 100 and 200 mM) were used to evaluate the possibility of improving enzymatic activity at pH 5.0, 6.0, and 7.0. This pH range covers the intestinal pH of larvae and adult bees [22] where the enzyme needs to be active. In fact, two feasible ways of administering the endolysin to larvae bees might be i) the incorporation in a sucrose solution to be supplied to adult bees that will subsequently feed the larvae; ii) spraying the suspension directly to larvae combs so that larvae can eat it.

**Fig 2:**
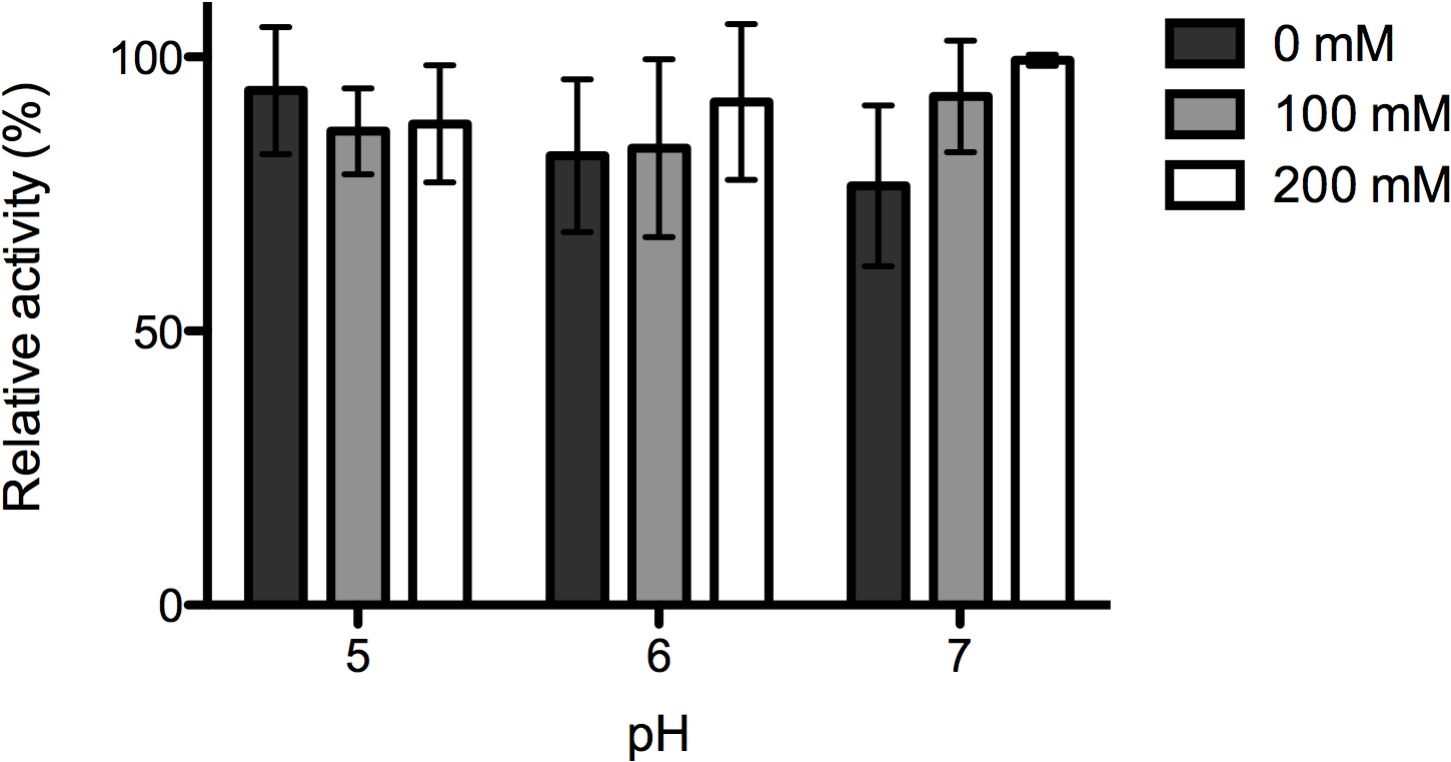
Effect of ionic strength on PlyPl23 lytic activity. PlyPl23 activity on *P*. *larvae* Pl02-23 at different pH values (pH 5.0, 6.0, and 7.0) supplemented with 0, 100 and 200 mM NaCl. Relative lytic activity was obtained as the ratio between the lytic activity of each test and the highest value obtained (the reference value was 50.4% lytic activity). Each column represents the mean of triplicate experiments and error bars indicate the standard deviation.

Statistical analysis revealed that for pH 5.0 and 6.0, no significant differences (p>0.05) were observed in the enzyme activity between any of the tested NaCl concentrations (Fig 2). For pH 7.0 the addition of 200 mM enabled a significantly higher activity compared with 0 mM (p<0.001) but the differences between 0 mM and 100 mM, and between 200 mM and 100 mM were not significant. Moreover, for pH 6.0 and 7.0 the average enzyme activity was higher with 200 mM NaCl. Based on these results, the antimicrobial activity of the enzyme was tested in pH 7.0 (matching the bee larvae intestine), supplemented with 200 mM NaCl.

#### Enzyme concentration and *in vitro* antimicrobial activity assay

**Fig 3:**
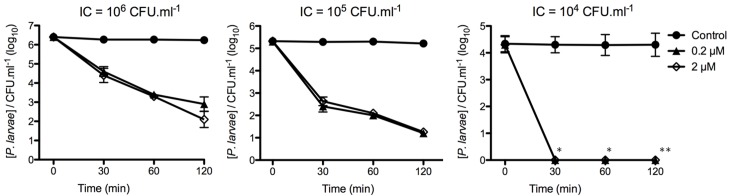
PlyPl23 activity on different *P*. *larvae* concentrations. PlyPl23 activity on *P*. *larvae* Pl02-23, at different enzyme (0.2 μm and 2.0 μm) and bacteria concentrations (10^6^, 10^5^ and 10^4^ CFU.mL^-1^). *Detection limit of 100 CFU.mL^-1^; ** Detection limit of 10 CFU.mL^-1^.

The minimum concentration of PlyPl23 acting against *P*. *larvae* was examined. Initial concentrations (IC) of the *P*. *larvae* strain Pl02-23, 10^4^, 10^5^, and 10^6^ CFU.mL^-1^, were tested. These IC are expected to be harmful to bee larvae, based on previous bioassays reporting high mortalities with an infectious *P*. *larvae* dose of 5x10^3^ CFU.mL^−1^ [28]. The results of the activity tests depicted in Fig 3 illustrates that for all tested bacterial IC, the use of either 0.2 μM or 2.0 μM PlyPl23 did not cause significant differences between them (p<0.001) on the bacterial reduction, in any of the 3 considered time periods, 30, 60 and 120 min. The activity of the enzyme in a 10^4^ CFU.mL^-1^ suspension allowed the reduction of cells to non-detectable levels just after 30 min. The limit of detection for the method applied was 100 CFU.mL^−1^ at 30 and 60 min, and 10 CFU.mL^−1^ at 120 min. When the enzyme was added to a 10^5^ CFU.mL^-1^ planktonic culture, after 30 and 60 min, an average log reduction of 2.8 and 3.3 log was observed, with an average decrease of 3.9 log after 120 min. For an IC of 10^6^ CFU.mL^-1^, the higher average reduction obtained after 120 min was 3.8 log in average, however the decrease observed was slower than with the IC 10^5^ CFU.mL^-1^: 1.7 and 2.8 log in average, respectively 30 and 60 min post-reaction.

#### Effect of sucrose, larval gut content and royal jelly

**Fig 4:**
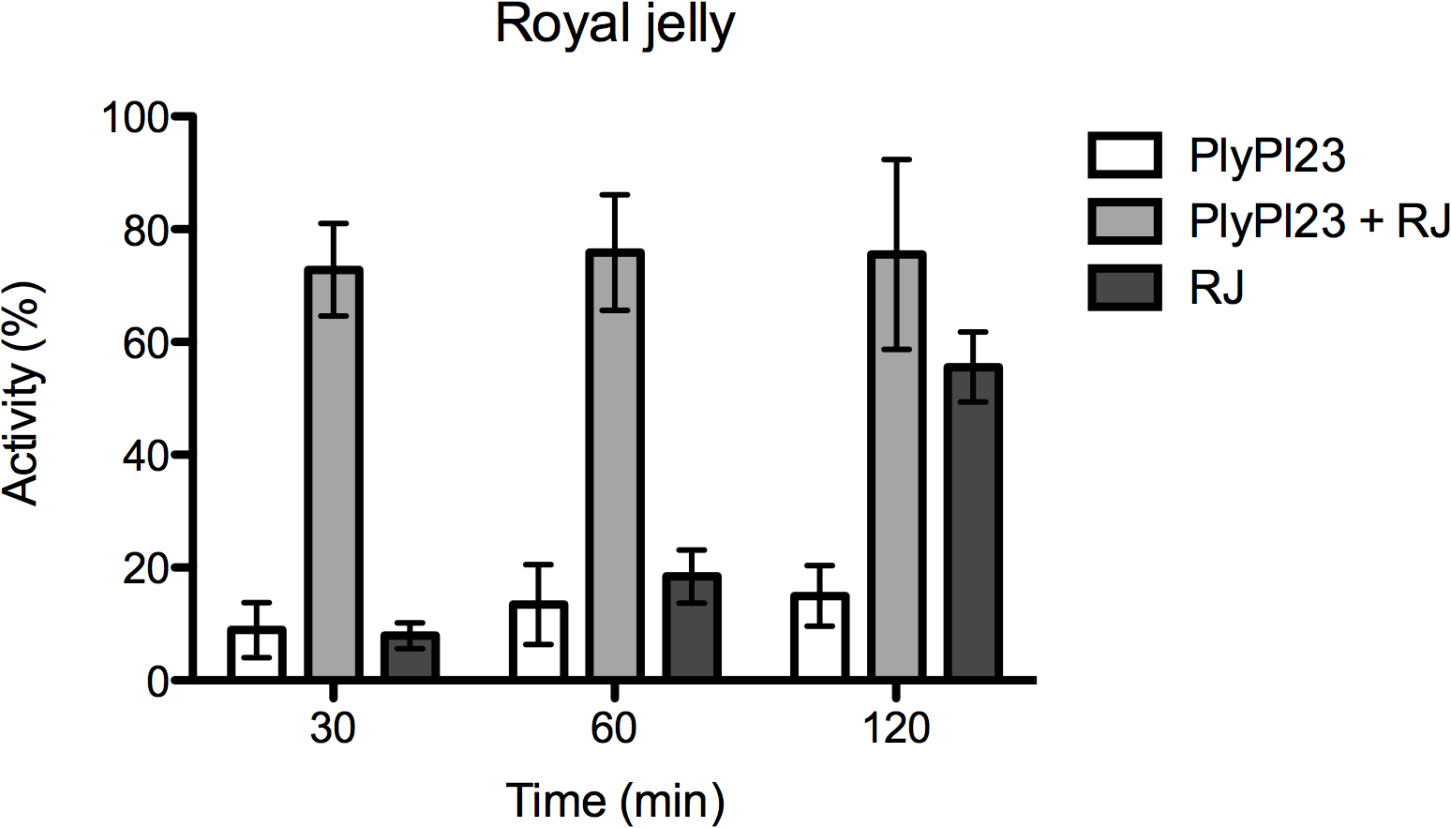
Effect of RJ on PlyPl23 activity. Activity of the enzyme after incubation in RJ for 1 h at 37°C. Results were recorded at 30, 60 and 120 min. Each column represents the mean of triplicate experiments and error bars indicate the standard deviation.

The effect of sucrose, HL and RJ on PlyPl23 activity was assessed, regarding the practical application. No differences in PlyPl23 activity after previous contact with 25% or 50% sucrose solution were observed. The enzyme activity in HL also remained unaffected in a 50% sucrose solution (prepared in 200 mM NaCl), supplemented with RJ (1:1 w/v) when incubated for 6 h at 37°C. Moreover, besides the enzyme was not inactivated, its activity was enhanced after previous contact with RJ, itself an antimicrobial substance (Fig 4). Results revealed a synergistic antimicrobial effect between PlyPl23 and RJ after 30 min and 60 min (the activity obtained for PlyPl23+RJ was 72.8% and 75.9%, respectively, higher than the addition of the activity of each component individually (30 min: 8.9% PlyPl23 and 7.9% RJ; 60 min: 13.5% PlyPl23 and 18.4% RJ), and an additive effect 120 min post-reaction (the activity of PlyPl23+RJ (75.6%) was statistically similar (p<0.001) to the activity of PlyPl23 (15.0%) + RJ (55.6%). It was also observed that the combination PlyPl23+RJ allowed a faster decrease in cell concentration, since the activity recorded 30 min after reaction with the combined antimicrobials was similar to the obtained after 120 min with the additive effect of the same substances, individually.

#### Development of resistances in *P*. *larvae* induced by PlyPl23

In order to evaluate the development of resistance in *P*. *larvae* induced by PlyPl23, a lower concentration of enzyme (MIC_50_) was used aiming at inducing resistant colonies. The experiment showed that the colonies recovered from the exposure at MIC_50_ of the endolysin were still sensitive to 2.0 μM of PlyPl23 (data not shown), even after ten cycles.

#### Evaluation of the stability of PlyPl23 and storage conditions

Results from the enzyme stability revealed that, when stored at 4°C and RT, the endolysin loses its activity after one and five weeks, respectively. The storage of PlyPl23 at -20°C for 22 weeks did not affect the enzyme activity (S2 Fig). The activity of the endolysin before lyophilization and after reconstitution remained the same (p>0.05) (data not shown).

#### Effect of PlyPl23 on spores

From the assays performed to examine the ability of PlyPl23 to degrade HA *P*. *larvae* spores with and without germinants, no OD_580_ variation (p>0.05) was observed during the experiment period (7 h) when compared to the control samples, where the endolysin was not present. The same was observed on samples where the endolysin was added 2 h after inducing germination. It was confirmed through OD_580_ variation that the germinants were able to induce spore germination (data not shown).

Microscopic observations enabled to verify differences on spore staining, between the sample collected at the time point 0 min and all the other samples (where only spores were observed, and predominantly red-pink).

#### Toxicity tests in artificially reared larvae

**Fig 5:**
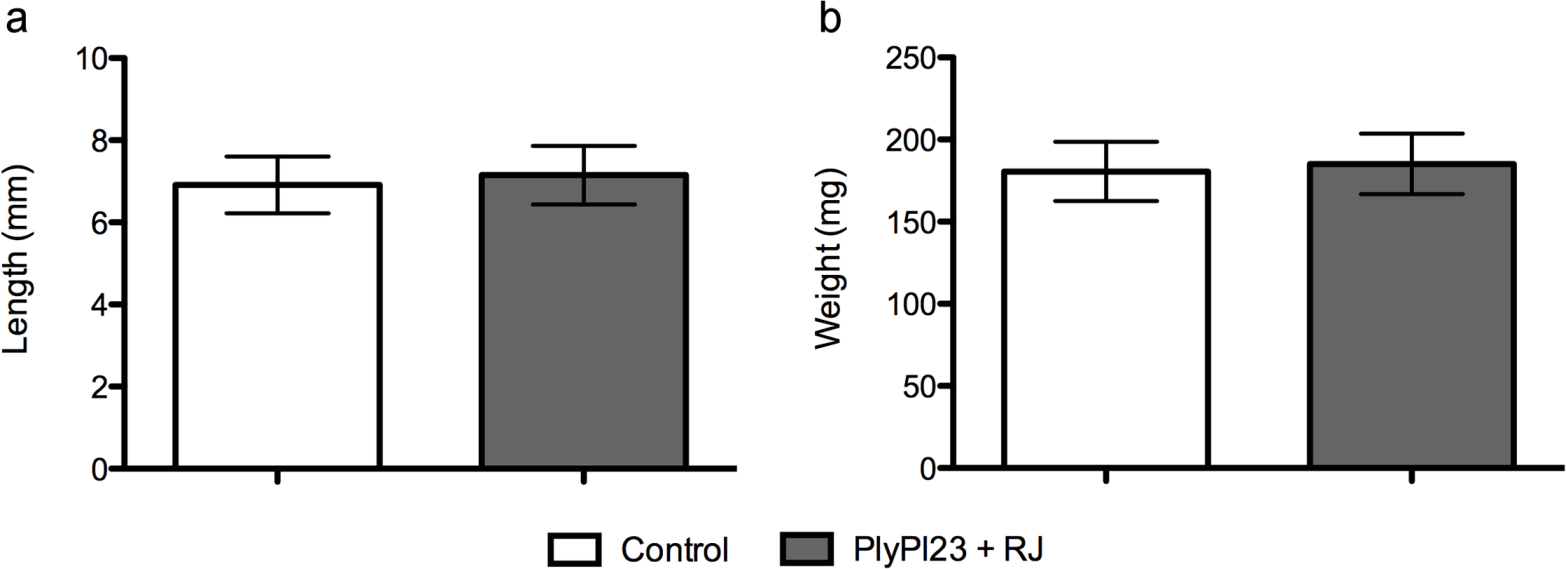
Variation in the larvae C-length and weight with PlyPl23 ingestion. Data presented refers to the increase of C-length (a) and larvae weight (b) between the 2^nd^ and the 5^th^ day. Comparison between the test group, that received diet supplemented with 2.0 μM PlyPl23 and the control group, receiving diet with buffer Tris pH = 8.0. Each column represents the mean of 13 larvae and error bars indicate the standard deviation (10%).

*In vivo* assays were performed to assess the potential toxic effect of the endolysin to bee larvae. The results demonstrated that larvae development was not affected by adding PlyPl23, as the C-length and weight of the larvae that were fed with diet supplemented with the enzyme, 7.1 cm and 185.2 mg respectively, did not differ from the control group (p>0.05), in average 6.9 cm and 180.6 mg, respectively (Fig 5). The estimation of the variability within the sample (measured by the standard deviation) was 10%.

## Discussion

*Paenibacillus larvae* spore germination and subsequent colonization of the young larval midgut (first- or second-instar larvae, occurring within about 48 h after egg hatching) is one of the key steps in the pathogenesis of these bacteria and consequently the critical factor to the onset of AFB [29]. To date, six *P*. *larvae* phage genomes were analyzed and compared [30]. From these phages, phiIBB_Pl23 is the only siphovirus and presents the lower genome size, being thus, the most distantly related genome from the other five myoviruses. Moreover, only in phiIBB_Pl23 was possible to identify a putative lysin.

In this study, the *in vitro* potential of the endolysin PlyPl23 to control the vegetative form of the Gram-positive bacterium *P*. *larvae* was assessed. This enzyme has an identifiable catalytic domain at the N-terminus, an *N*-acetylmuramoyl-*L*-alanine amidase. It was not possible to identify *in silico* any domain in the C-terminus of the enzyme peptide sequence. Since the endolysin is from a Gram-positive background, the existence of a novel CBD can be hypothesized. In order to evaluate the endolysin spectrum of activity, several field strains were isolated and identified as *P*. *larvae* subsp. Larvae, based on its 16S rRNA [11] and rep-PCR [8]. While the phage phiIBB_Pl23 showed to be active against 80% of the strains, PlyPl23 lysed 100% of the isolated strains. Both are active against the most relevant genotypes ERIC I (more prevalent in Europe) and ERIC II (only identified in Europe), as ERIC III and IV have not been isolated for many years [4]. Other endolysins demonstrating broader lytic spectra when compared with the host range of the respective phage were already reported [31]. The high lytic efficiency is also supported by the hypothesis that amidases generally display a broader spectrum of antibacterial activity than the other classes of endolysins due to the very frequent occurrence of the amide bond between *N*-acetylmuramic acid and *L*-alanine in peptidoglycan [32]. Nevertheless, PlyPl23 was not active against the other tested species of *Bacillus* and *Lactobacillus*. This supports the potential broad application of a PlyPl23-based product specifically against AFB. For *in vivo* application purposes, the most feasible way of administering an enzyme-based product to control AFB is by oral administration to adult bees or by spraying directly to bee larvae. Thus, besides exploring the best activity conditions for PlyPl23, the performance of the enzyme in conditions envisaging the practical application were also tested. Accordingly, the activity tests were performed at 37°C (which is similar to the hive temperature, about 34–35°C) and particular attention was given to pH 5.0 to 7.0,which includes the pH of the bee larvae intestine, pH 6.8, and adult bees pH 5.6 to 6.3 [22]. Furthermore, the effect of HL, sucrose and RJ on PlyPl23 activity was also tested. These are the media that better simulate a potential *in vivo* application. The contact of PlyPl23 with sucrose intended to simulate the incorporation of the enzyme in food, since sucrose is used routinely as an artificial source of sugar for bees [33]. The tolerance of PlyPl23 to RJ (a nutritive and antimicrobial substance secreted by nurse bee hypopharyngeal gland) was assessed, since the spray administration would promote the contact between both substances previous to injestion by the larvae. In fact, bee larvae float on RJ since egg hatching, using it as food source until the early larval stage (up to 2.5 to 3 day old) [20, 34]. Finally, HL was used to simulate the gastrointestinal conditions of the larvae, where the enzyme will have its major antimicrobial activity.

The *in vitro* evaluation of the enzyme activity showed very promising results. In fact, it was found that the enzyme displayed high antimicrobial activity at pH 5.0 to 9.0, which includes the pH values of interest (5.0 to 7.0). Furthermore, PlyPl23 remained active in HL, after contact with 25% and 50% sucrose. In addition, and interestingly, PlyPl23 activity was enhanced after contact with RJ.

The reason of the synergistic effect is unknown, however, the broad-spectrum distribution of substances in RJ [35] might suggest that some of them can be acting as cofactors. Besides, RJ might sensitize the bacteria cell wall, allowing the easier and faster action of the endolysin. In fact, RJ alone has antimicrobial effect, which was already shown by other authors [36, 37]. However, the antimicrobial activity of RJ is significantly slower than its combination with PlyPl23.

Subsequent tests assessed the *in vitro* antimicrobial capacity of PlyPl23. It was demonstrated that 0.2 μM PlyPl23 (in Tris pH 7.0 (20 mM), supplemented with 200 mM NaCl) was able to decrease a population of 10^4^ CFU.mL^-1^ of *P*. *larvae* to non-detectable levels (< 10 colonies). Higher bacterial concentrations (10^5^ CFU.mL^-1^ and 10^6^ CFU.mL^-1^) of *P*. *larvae* exposed to the same enzyme concentration were consistently reduced by approximately 4-log units. Based on previous reports of exposure bioassays in artificially reared honey bee larvae [28], it can be inferred that a concentration of 5x10^3^ CFU.mL^−1^ viable *P*. *larvae* cells are enough to cause the death of about 70% of the infected larvae. Consequently, the results obtained showed that PlyPl23 is highly efficient in controlling bacterial loads of *P*. *larvae* commonly found in infected hives.

Besides the already discussed enzyme features, studies aiming to assess the ability of the enzyme to induce resistance revealed that after ten cycles of exposure to sub-inhibitory concentrations (MIC_50_), the bacteria remained susceptible to the enzyme. These results indicated that *P*. *larvae* was not able to acquire resistance to PlyPl23. Similar results were reported for other endolysins of Gram-positive background [21, 38]. This emphasizes the value of a potential product when aiming an *in vivo* application.

The enzyme is very stable when stored at -20°C (similar results for other proteins were already reported by (Turck and Bierbaum [39]) still stable at 4°C, but it looses activity at RT. Nevertheless, the activity was preserved when the endolysin was lyophilized indicating that storage of a PlyPl23-based product may be feasible, turning it in an economically viable product.

The results have shown that the *P*. *larvae* spores were not sensitive to PlyPl23. The endolysin was inactive against both dormant and germinating spores (at time 0h and 2h after induction). This suggests that the increased porosity of the spore proteinaceous coat naturally occurring on germinating spores does not enable the access of PlyPl23 to the peptidoglycan in contrast to what was already reported with an endolysin against germinating *Bacillus cereus* spores [21]. The insensitivity of the germinating spores at an early stage of germination reveals that the endolysin might act inside the larvae gut, which naturally induces spore germination, but only after germination is complete.

Furthermore, the enzyme administration to artificially raised larvae did not impair the normal growth and weight gain of young larvae, indicating that it is not toxic for the larvae.

The present work describes, to our knowledge, the first characterization of an endolysin from a *P*. *larvae* phage. Overall, the results obtained highlight the high potential of this protein in controlling *P*. *larvae* concentrations commonly found inside honeybee larvae in field conditions. Furthermore, there were no activity losses in conditions that mimic the hive environment where the endolysin must be administered and active. This encourages further *in vivo* experiments and reveals the high potential of PlyPl23 to integrate a commercial product to control the problematic AFB.

## Supporting Information

S1 Fig SDS-PAGE gel loaded with different fractions resulting from PlyPl23 purification. M- Protein marker; 1-Flow through; 2- wash with 20 mM imidazole; 3, 4, 5 – sequentially eluted fractions in 300 mM imidazole.
(TIF)

S2 Fig Evaluation of the stability of PlyPl23, after storage at different temperatures. Activity was tested after storage at 4°C, RT (room temperature) and -20°C for 22 weeks.
(TIF)
