# Correction: The First *Paenibacillus larvae* Bacteriophage Endolysin (PlyPl23) with High Potential to Control American Foulbrood

**DOI:** 10.1371/journal.pone.0136331

**Published:** 2015-08-14

**Authors:** Ana Oliveira, Marta Leite, Leon D. Kluskens, Sílvio B. Santos, Luís D. R. Melo, Joana Azeredo

The Data Availability statement for this paper is incorrect. The correct statement is: All relevant data are within the paper and its Supporting Information files, except the aminoacidic sequence of the endolysin, which is available in RefSeq, accession number YP_008320357.1.
