# Supplementary figures and images for: The First Paenibacillus larvae Bacteriophage Endolysin (PlyPl23) with High Potential to Control American Foulbrood
Source: PLoS One. 2015 Jul 13;10(7):e0132095. doi: 10.1371/journal.pone.0132095 (PMC4500393; doi:10.1371/journal.pone.0132095)

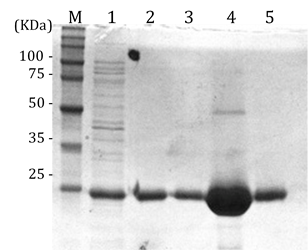

Supplement: S1 Fig — M- Protein marker; 1-Flow through; 2- wash with 20 mM imidazole; 3, 4, 5 – sequentially eluted fractions in 300 mM imidazole. (TIF) [file pone.0132095.s001.tif]

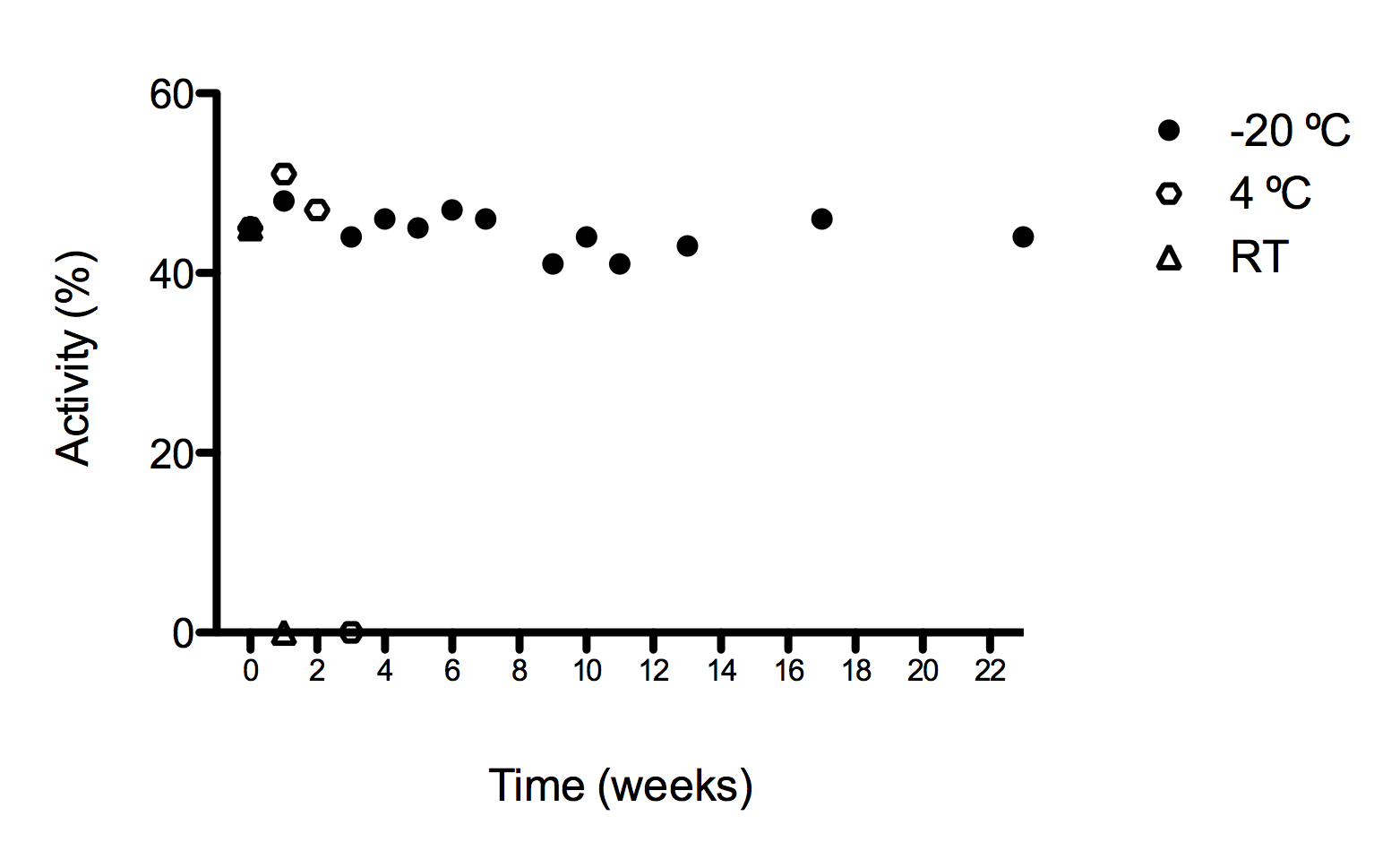

Supplement: S2 Fig — Activity was tested after storage at 4°C, RT (room temperature) and -20°C for 22 weeks. (TIF) [file pone.0132095.s002.tif]
